# Multi-Omics Identification of Genetic Alterations in Head and Neck Squamous Cell Carcinoma and Therapeutic Efficacy of HNC018 as a Novel Multi-Target Agent for c-MET/STAT3/AKT Signaling Axis

**DOI:** 10.3390/ijms241210247

**Published:** 2023-06-16

**Authors:** Harshita Nivrutti Khedkar, Lung-Ching Chen, Yu-Cheng Kuo, Alexander T. H. Wu, Hsu-Shan Huang

**Affiliations:** 1 Ph.D. Program for Cancer Molecular Biology and Drug Discovery, College of Medical Science and Technology, Taipei Medical University, and Academia Sinica, Taipei 11031, Taiwan; d621108005@tmu.edu.tw; 2 Graduate Institute for Cancer Biology & Drug Discovery, College of Medical Science and Technology, Taipei Medical University, Taipei 11031, Taiwan; 3 Division of Cardiology, Department of Internal Medicine, Shin Kong Wu Ho-Su Memorial Hospital, Taipei 11101, Taiwan; marcus1831@gmail.com; 4 School of Medicine, Fu Jen Catholic University, New Taipei 24205, Taiwan; 5 Department of Pharmacology, School of Medicine, College of Medicine, Taipei Medical University, Taipei 11031, Taiwan; yucheng.kuo@msa.hinet.net; 6 School of Post-Baccalaureate Chinese Medicine, College of Chinese Medicine, China Medical University, Taichung 40402, Taiwan; 7 Ph.D. Program for Translational Medicine, College of Medical Science and Technology, Taipei Medical University, Taipei 11031, Taiwan; 8 Taipei Heart Institute (THI), Taipei Medical University, Taipei 11031, Taiwan; 9 Clinical Research Center, Taipei Medical University Hospital, Taipei Medical University, Taipei 11031, Taiwan; 10 International Ph.D. Program for Translational Science, College of Medical Science and Technology, Taipei Medical University, Taipei 11031, Taiwan; 11 Graduate Institute of Medical Sciences, National Defense Medical Centre, Taipei 11490, Taiwan; 12 School of Pharmacy, National Defense Medical Centre, Taipei 11490, Taiwan; 13 Ph.D. Program in Drug Discovery and Development Industry, College of Pharmacy, Taipei Medical University, Taipei 11031, Taiwan

**Keywords:** bioinformatics, target-based structure discovery, molecular docking, head and neck squamous cell carcinoma, drug-likeness, drug resistance, multi-target therapeutics

## Abstract

Amongst the most prevalent malignancies worldwide, head and neck squamous cell carcinoma (HNSCC) is characterized by high morbidity and mortality. The failure of standard treatment modalities, such as surgery, radiotherapy, and chemotherapy, demands the need for in-depth understanding of the complex signaling networks involved in the development of treatment resistance. A tumor’s invasive growth and high levels of intrinsic or acquired treatment resistance are the primary causes of treatment failure. This may be a result of the presence of HNSCC’s cancer stem cells, which are known to have self-renewing capabilities that result in therapeutic resistance. Using bioinformatics methods, we discovered that elevated expressions of MET, STAT3, and AKT were associated with poor overall survival in HNSCC patients. We then evaluated the therapeutic potential of our newly synthesized small molecule HNC018 towards its potential as a novel anticancer drug. Our computer-aided structure characterization and target identification study predicted that HNC018 could target these oncogenic markers implicated in HNSCC. Subsequently, the HNC018 has demonstrated its anti-proliferative and anticancer activities towards the head and neck squamous cell carcinoma cell lines, along with displaying the stronger binding affinities towards the MET, STAT3, and AKT than the standard drug cisplatin. Reduction in the clonogenic and tumor-sphere-forming ability displays HNC018’s role in decreasing the tumorigenicity. Importantly, an vivo study has shown a significant delay in tumor growth in HNC018 alone or in combination with cisplatin-treated xenograft mice model. Collectively with our findings, HNC018 highlights the desirable properties of a drug-like candidate and could be considered as a novel small molecule for treating head and neck squamous cell carcinoma.

## 1. Introduction

Head and neck squamous cell carcinoma (HNSCC) is one of the most severe and challenging malignancies, with a high level of heterogeneity and a wide range of treatment responses, regardless of the clinical stage [1]. Lymph node metastasis is typically identified too late in the course of a patient’s illness. Thus, an early diagnosis of head and neck cancer is crucial. With a current global prevalence of 18.1 million, this type of cancer is the second greatest cause of death, and it is expected to increase by 75 percent to about 25 million cases by 2035 [2]. By 2025, it is expected that cancer-related mortality in less developed countries will have increased by 80% [2]. In head and neck cancers, a variety of genetic mutations play a role. Additionally, many environmental factors, such as HPV infection and alcohol and tobacco exposure, are associated with HNSCCs (Supplementary Figure S1). To date, the best treatment for HNSCC patients is a multimodal strategy that includes surgery, chemotherapy, radiation therapy, and systemic medicines [3,4,5]. The overall survival rates for patients with advanced stage HNSCC have remained low [3,6], within 24 months of treatment, and more than half of patients develop recurrence at distant or adjacent site [7,8]. Head and neck cancer patients have high morbidity and poor quality of life due to surgical failure, resistance to adjuvant radiotherapy and chemotherapy, and post-therapy complications, such as renal and mucosal impairments, necrosis, immune suppression, muscle fibrosis, and mandibular fractures [7,9]. Due to the intrinsic and acquired drug resistance and limited medication efficacy, targeted treatments have only benefited a small subset of patients [10,11,12,13,14]. Head and neck cancer comprises a diverse group of extremely aggressive tumors, and is made up of many different subsets of cells that infiltrate the tumors and interact with tumor cells or with each other via multiple networks [15]. 

A receptor tyrosine kinase (RTK), mesenchymal–epithelial transition factor, is encoded by the c-MET proto-oncogene on chromosome 7q21-q31 [16,17,18]. c-MET has numerous functional domains, including the juxtamembrane (JM) (regulatory domain), the receptor tyrosine kinase domain (TK), and the semaphoring (SEMA) domain, which binds to its ligand, the hepatocyte growth factor (HGF) [19]. The binding of c-MET to HGF causes c-MET dimerization, auto-phosphorylation, and activation of TK catalytic activity, which activates other cellular signaling pathways, such as RAS/RAF/ERK, PI3K/AKT/mTOR, JAK/STAT, and NOTCH, resulting in cell growth, motility, and survival [17,20]. Overexpression of solid tumors was seen in 57% of SCC, resulting in increased cell motility, stemness, invasion, and a poor prognosis [21,22]. Simultaneously, IL-6/STAT3 signaling in malignant EMT leads to the acquisition of cancer stemness in cancer cells; self-renewal and population expansion of CSCs need STAT3 in collaboration with stem-cell-associated transcription factors [23]. STAT3 is crucial for controlling CSCs of cancers such ovarian cancer [24], HCC [25], breast cancer [26], colorectal cancer [27], glioblastoma [28], lung cancer [29], and prostate cancer [30], given its significant function in preserving the self-renewal and differentiation of embryonic stem cells (ESCs) [31,32,33]. The regulation of stem cell self-renewal and differentiation by STAT3 focuses mostly on the ESC-specific functions of the Leukemia inhibitory factor (LIF) [31]. 

A number of key routes have been related to HNSCC resistance mechanisms. Growth factors or cytokines activate the AKT/PI3K pathway by activating an external receptor, such as a receptor tyrosine kinase (RTK) or G-protein coupled receptor. The signal is delivered intracellularly via the enzymatic activation of phosphatidylinositol (3, 4, 5) triphosphate (PIP3) by phosphoinositide 3-kinase (PI3K). PIP3 subsequently activates protein kinase B (AKT), which phosphorylates a variety of other proteins involved in cell cycle progression, survival, and growth [34]. One intriguing study indicated that HNSCC cells resistant to cisplatin grew slower when treated with a MEK inhibitor plus afatinib. The MEK inhibitor suppressed the RAS/RAF/MEK/ERK pathway and produced enhanced AKT signaling, while afatinib synergistically blocked HER family signaling and the AKT pathway [35].

**Figure 1:**
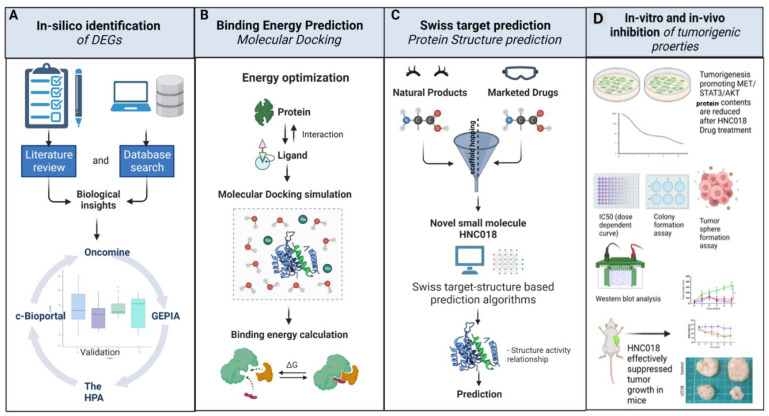
Schematic representation of the study. Column (**A**) represents the multi-omics identification of differentially expressed genes from various online databases. Column (**B**) represents binding energy prediction analysis achieved by performing molecular docking with specific inhibitor and differentially expressed genes. Column (**C**) represents synthesis of new molecule through scaffold hopping and target-based structure discovery of potential druggable candidates for HNC018 identified through Swiss target prediction online web portal. Column (**D**) represents the in vitro and in vivo therapeutic efficacy analysis performed with HNC018 as a potential anticancer agent.

Preclinical trials of mono-therapeutic drugs with distinct mechanisms of action found it challenging to overcome HNSCC treatment resistance [3]. Based on these necessities, a small molecule drug newly synthesized in our lab, HNC018, was evaluated for its anticancer properties; Figure 1 indicates the schematic representation of the study. Herein, we provide in vitro and in vivo evidence that HNC018 exhibits anti-tumor effects by suppressing the c-MET/STAT3/AKT signaling axis. We discovered higher expression of c-MET, STAT3, and AKT in HNSCC patients with a poor prognosis and malignant phenotype using bioinformatics methods. Consequently, the medicinal potential of a novel small molecule, HNC018, was then assessed by in vitro studies. HNC018 had strong interactions with oncogenic molecules, such as c-MET, STAT3, and AKT, according to a molecular docking study. HNC018 therapy inhibited HNSCC tumorigenicity and reduced the cancer stem cell-like properties by inhibiting the growth of tumor spheres, as well as cisplatin resistance in an in vitro study. HNC018 treatment alone inhibited tumor growth in an HNSCC cell line mouse xenograft model, and when paired with cisplatin, it had a stronger inhibitory activity. In addition, HNC018 therapy significantly lowered the expression of c-MET, STAT3, and AKT and their phosphorylated forms in the in vitro study and showed better binding affinities than standard medication in a molecular docking analysis, eliciting its anticancer effect. Taken together, our data support the development of HNC018 as a multi-targeted novel small molecule for head and neck squamous cell carcinoma.

## 2. Results

### 2.1. Discovery of a Novel Small Molecule, HNC018, via Scaffold-Hopping of Bioactive Compounds

**Figure 2:**
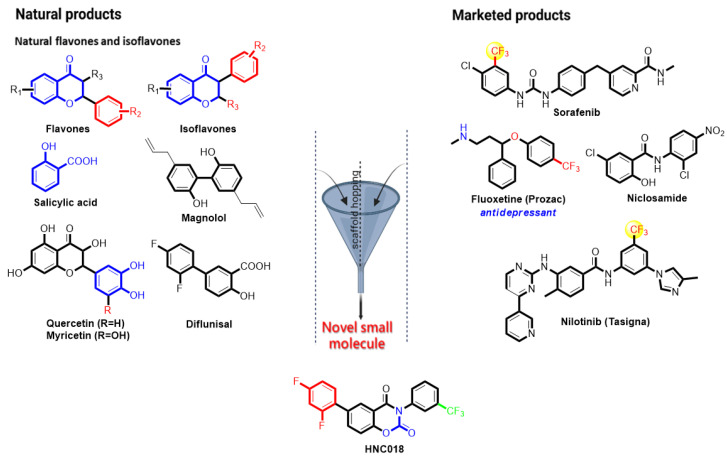
Scaffold hopping of bioactive natural compounds (flavones and iso-flavones), biphenyl-tri-fluoro-methyl-phenyl, and niclosamide lead to the discovery of a novel small molecule.

The use of bioactive compounds in multiple scaffolds is a crucial method for creating novel medications [36]. The important natural product backbones include biphenyl, flavones, and isoflavones. A number of bioactive compounds containing these backbones have been reported to have a wide range of biological effects, including those that are anti-oxidative, anti-atherosclerotic, muscle-relaxing, anti-microbial, anti-inflammatory, and anticancer [37,38]. An essential moiety that has been linked to the effects of several medications is trifluoromethylphenyl (Figure 2; template created with BioRender.com, accessed on 8 March 2022). Trifluoromethylphenyl is a key ingredient that contributes to the bioactivity of a number of clinical medications, including nilotinib (a tyrosine kinase inhibitor with antineoplastic activity), fluoxetine (an anti-depressant, anti-obsessional, anti-anxiety, and immune-modulating agent), and sorafenib (an RAF/MEK/ERK inhibitor with antineoplastic activity). Niclosamide is a versatile drug with a track record of success in the treatment of a number of illnesses, including cancer, oxidative stress, infections, metabolic disorders, and inflammation [39,40,41,42,43,44,45,46]. A scaffold-hopping (Figure 2) of these bioactive natural chemicals (flavones and isoflavones), biphenyl, trifluoromethyl, and niclosamide led to the development of a novel multi-target small molecule in the present study: 6-(2, 4-difluorophenyl)-3-(3-(trifluoromethyl) phenyl)-2H-benzol (e) (1, 3) oxazine-2, 4 (3H)-dione (HNC018). Next, we investigated the compound’s potential as a therapeutic target against head and neck cancer-hub genes using an in silico ligand–receptor interaction analysis (Supplementary Figures S2 and S3, Supplementary Table S1).

### 2.2. Frequent Overexpression of MET/STAT3/AKT Genes Observed across the Oncomine Database in HNSCC

**Figure 3:**
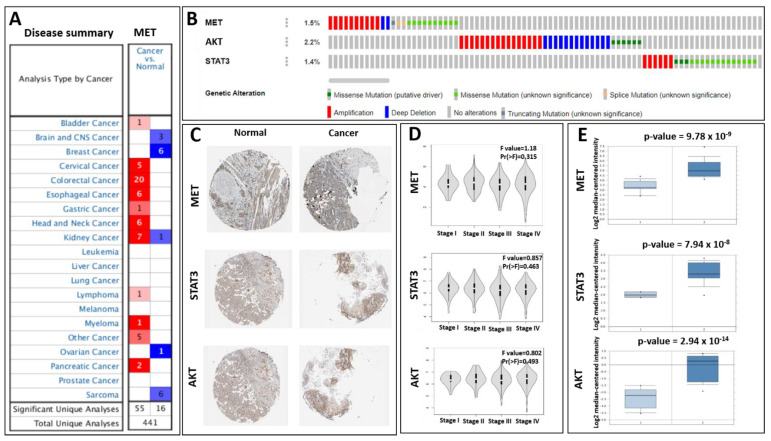
Expression profiles of the MET, STAT3, and AKT genes in head and cancer in multiple databases. (**A**) Utilizing the Oncomine database, the expression of MET, STAT3, and AKT in head and neck cancer and normal tissue samples was analyzed. MET is overexpressed in a variety of malignancies, notably in patients with head and neck cancer, according to the findings. (**B**) c-BioPortal study of MET, STAT3, and AKT gene mutations. The c-BioPortal cancer genomics database was used to assess MET, STAT3, and AKT gene mutations in head and neck cancer using an OncoPrint bar code plot. (**C**) Expressions of MET, STAT3, and AKT in head and neck cancer tissues and normal tissues in the Human Protein Atlas. (**D**) Correlations between the expressions of MET, STAT3, and AKT with the tumor stage in patients with head and neck cancer. From the GEPIA database, correlations of MET, STAT3, and AKT expressions with the tumor stage in patients with head and neck cancer were used to generate a violin plot; the *p*-value was set to 0.05. The abscissa represents the stage of head and neck cancer, while the ordinate represents MET, STAT3, and AKT expression levels. (**E**) Expressions of MET, STAT3, and AKT on chips from various head and neck cancer research studies contained in the Oncomine database.

The Oncomine database was utilized to analyze the expression of MET, STAT3, and AKT in head and neck cancer and normal tissue samples. The findings revealed that MET is over-expressed in a variety of malignancies, particularly in patients with head and neck cancer. (Figure 3A). We mined the TCGA’s head and neck cancer genomics data using the c-BioPortal website to study the roles of MET, STAT3, and AKT overexpression [47,48]. Amplification, deep deletion, and truncating mutations were found in patients’ genomic data, and these patients had increased mRNA levels of MET, STAT3, and AKT expression (Figure 3B). In addition, the Human Protein Atlas database was utilized to confirm the histological levels of MET, STAT3, and AKT, and the results indicate that these genes were elevated in head and neck cancer tissue relative to normal tissue (Figure 3C). Intriguingly, we discovered that the expressions of MET, STAT3, and AKT were linked with the tumor stage using the web-based application GEPIA (Figure 3D). This suggests that they play essential roles in the tumorigenesis of head and neck cancer. The TCGA head and neck cancer statistics were filtered, and it was discovered that MET, STAT3, and AKT gene expressions were greater than those in the control group (Figure 3E).

### 2.3. Genetic Alterations of MET Are Associated with Poor Prognoses of Cancer Patients

**Figure 4:**
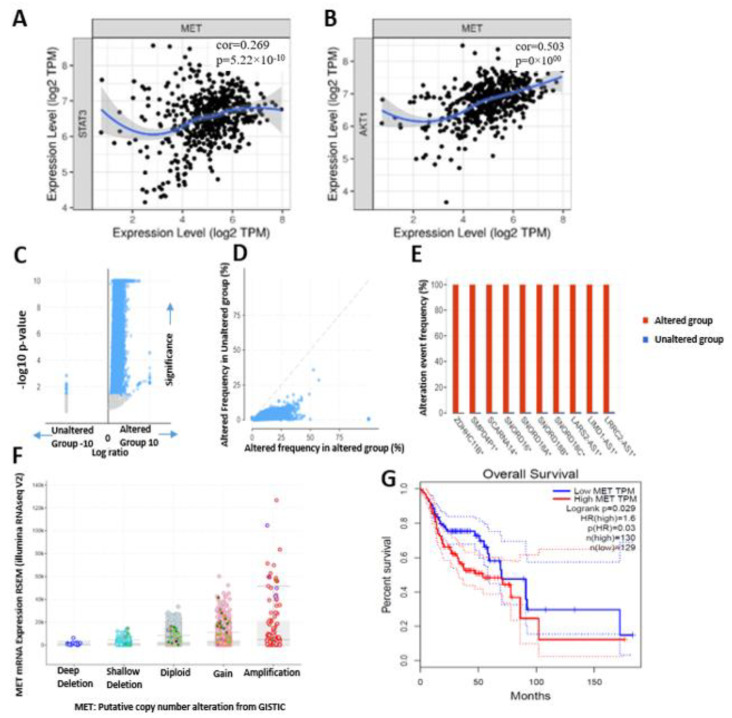
Correlations analysis and genomic alterations in MET are associated with poor prognosis of cancer cohorts. (**A**,**B**) Expression scatterplots of MET correlations with STAT3 and AKT in head and neck cancer. The strength of correlations between the genes is reflected by the purity-adjusted partial Spearman’s rho value, where a value of r = 1 means a perfect positive correlation and a value of r = −1 means a perfect negative correlation. (**C**) Heat map showing *p*-values and significance levels of other gene mutations associated with cohorts with altered MET and cohorts with unaltered MET. (**D**) Line graph showing frequencies of altered genes in cancer cohorts with altered MET and cohorts with unaltered MET. (**E**) Bar graph showing gene mutations that were significantly enriched in both MET-altered and MET-unaltered cohorts. (**F**) Bar showing the MET putative copy number alterations and messenger RNA expressions. (**G**) Cohorts with altered MET had low overall survival (*p* value = 0.029).

In head and neck cancer, MET expression was significantly correlated with STAT3 (r = 0.269) and AKT (r = 0.503). Scatterplots of MET’s correlation with STAT3 and AKT are given in Figure 4A,B. The strength of the correlations between the genes is reflected by the purity-adjusted partial Spearman’s rho value, where a value of r = 1 means a perfect positive correlation and a value of r = −1 means a perfect negative correlation. We also determine the gene alteration co-occurrence frequencies with respect to MET-altered and MET-unaltered cohorts and found a number of other gene mutations that co-occurred with MET (Figure 4C,D). The most common alterations of MET were amplification and gene gain, while gene deletions were the least common alterations in the MET genes (Figure 4F). The top ten gene mutations that were significantly (*p*-value 1.23 × 10^−163^ to 9.57 × 10^−151^ and q-value 1.01 × 10^−110^ to 9.17 × 10^−144^) enriched in the MET-altered cohorts were CAPZA2, ST7, TES, WNT2, ASZ1, CFTR, TFEC, CTTNBP2, CAV2, CAV1, MDFIC, ANKRD7, and FOXP2 (Figure 4C and Table 1). Meanwhile, WASH5P, LINC01002, C190RF25, STARD4-AS1, and LINC02200 (*p*-value 1.40 × 10^−03^ to 7.00 × 10^−03^ and q-value 1.68 × 10^−03^ to 7.93 × 10^−03^) were enriched in unaltered MET cohorts (Figure 4D, Table 2). However, 10 genes, including ZDHHC11B (100%, 0.19%), SMPD4P1 (100%, 0.29%), SCARNA14 (100%, 0.50%), SNORD16 (100%, 0.51%), SNORD18A (100%, 0.51%), SNORD18B (100%, 0.51%), SNORD18C (100%, 0.51%), LARS2-AS1 (100%, 0.67%), LIMD1-AS1 (100%, 0.69%), and LRRC2 (100%, 0.77%), had higher mutation frequencies in the MET-altered cohort than the unaltered cohort, respectively (Figure 4E). These alterations in the MET genes were associated with low overall survival (*p*-value < 0.029) (Figure 4G).

### 2.4. HNC018 Treatment Inhibits Tumorigenesis and Cancer Stem Cell-like Properties via Downregulating c-MET/STAT3/AKT Expressions

**Figure 5:**
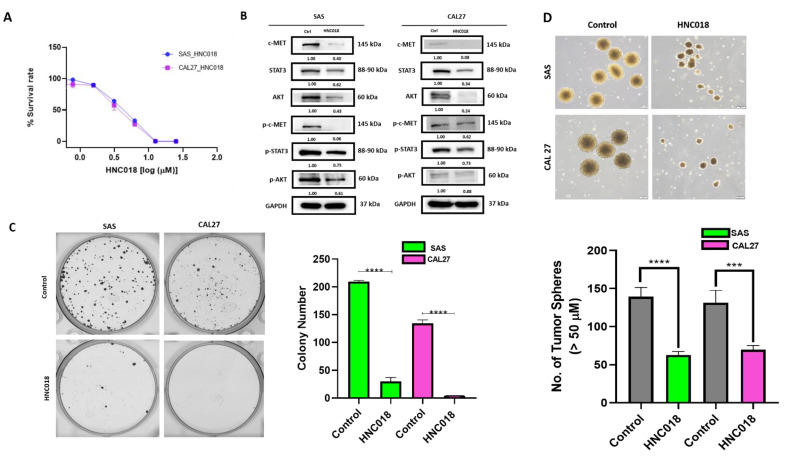
HNC018 treatment inhibited tumorigenic and stemness properties via downregulating the c-MET/STAT3/AKT expression. (**A**) HNC018 treatment resulted in a dose-dependent reduction in cell viability in both SAS and CAL27 cells. (**B**) Western blot of HNC018-treated SAS and CAL27 cells exhibited lower expression levels of c-MET, p-c-MET, STAT3, p-STAT3, AKT, and p-AKT. HNC018 treatment significantly reduced the colony formation in SAS and CAL27 (3.6 µM and 3.1 µM, respectively) (**C**) and tumor-sphere forming abilities (**D**) in both SAS and CAL27 (11.7 µM and 9.8 µM, respectively). *p <* 0.001 (***), and *p* < 0.0001 (****).

After finding that c-MET plays a part in the tumorigenicity of HNSCC by enhancing stem cell-like phenotypes [49,50,51], we looked into the potential inhibitory effect and therapeutic effectiveness of HNC018 on the SAS and CAL27 HNSCC cell lines in particular. We demonstrated that low dose HNC018 dramatically reduced the viability of SAS and CAL27 HNSCC’s cells in a dose-dependent manner using the SRB colorimetric assay (Figure 5A). We found that the SAS and CAL27 HNSCC’s cells had IC50 values of 4.1 µM and 3.5 µM, respectively. HNC018 dose-dependently reduced the cell viability in both the SAS and CAL27 cells. As this is a dose-dependent assay, we used the drug inhibition concentration 40 (i.e., IC40 concentration) to further perform a colony formation and a Western blot assay, to determine the efficacy of the drug at a lower concentration. We examined the impact of HNC018 on the biologic characteristics of the HNSCC cells because the ability to establish colonies in distant anatomic areas is essential for the progression and recurrence of HNSCC. The HNC018-treated cells showed significantly reduced abilities to form colonies and reduced tumor spheres in both the SAS and CAL27 cell lines. Using Western blot assays, we assessed the expression of our chosen panel of oncogenic proteins to better understand the underlying mechanism for the HNC018-mediated decrease in HNSCC cell viability (Figure 5A), clonogenicity (Figure 5C), and tumor sphere formation (Figure 5D). Furthermore, we found that HNC018 treatment (3.6 and 3.1 µM for the SAS and CAL27 cell lines, respectively) (Figure 5B, Supplementary Figure S4 and Supplementary Table S2) significantly reduced the expression level of c-MET total protein and its phosphorylated forms p-c-MET, and this was linked to the simultaneous downregulation of STAT3, p-STAT3, AKT, and p-AKT (Figure 5B).

### 2.5. HNC018 Synergistically Enhances the Anticancer Effect of Cisplatin in Therapy-Resistant HNSCC Cells

**Figure 6:**
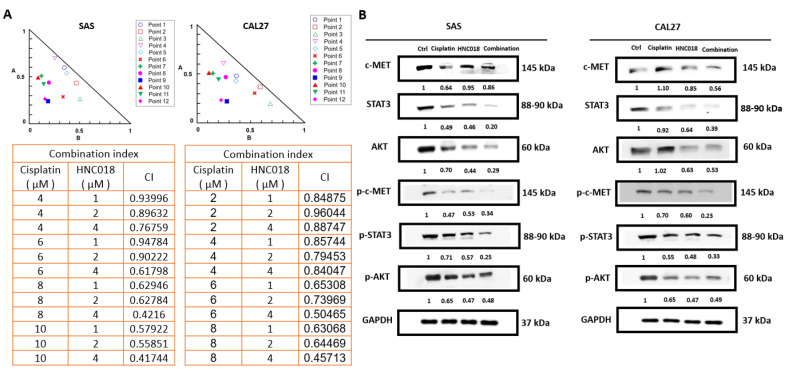
HNC018 improves the anticancer impact of cisplatin in HNSCC cells resistant to therapy. (**A**) The cisplatin-HNC018 combination points with the indicated concentrations and CIs in HNSCC cells are shown in representative right-angle isobologram triangles. (**B**) Western blot results demonstrating how HNC018 and cisplatin affect the level of c-MET, p-c-MET, STAT3, p-STAT3, AKT, and p-AKT protein expression in HNSCC cells as compared to control cells.

Considering that cisplatin-resistance is a challenge in the selection of therapy for patients with HNSCC and that HNC018 demonstrates significant anti-stem cell effects, we investigated whether HNC018 could be combined with cisplatin and examined the effect of such a combination on the anticancer activity of cisplatin. Treatment with 4–10 µM cisplatin and 1–4 µM HNC018 successively reduced the viability of the SAS and CAL27 cells, demonstrating that the anticancer effects of cisplatin were enhanced. We found that all the drug combination points were within the right-angle isobologram triangle and that all the CI values were <1, indicating that the combined action of HNC018 and cisplatin against the SAS and CAL27 cells was synergistic, using the Chou–Talalay-based algorithm for drug combination analysis (Figure 6A, Supplementary Figures S5 and S6). Our results suggest that HNC018 synergistically improves cisplatin’s anticancer effectiveness in HNSCC cells. Cisplatin–HNC018 combination therapy significantly reduced the expression levels of c-MET, p-c-MET, STAT3, p-STAT3, AKT, and p-AKT compared to the levels when treated with cisplatin or HNC018 alone (Figure 6B, Supplementary Figure S7). According to these findings, HNC018 synergistically improves cisplatin’s anticancer effectiveness in HNSCC cells.

### 2.6. PPI Clustering Network Revealed Multiple Interactions of MET/STAT3/AKT with Oncogenic Proteins

**Figure 7:**
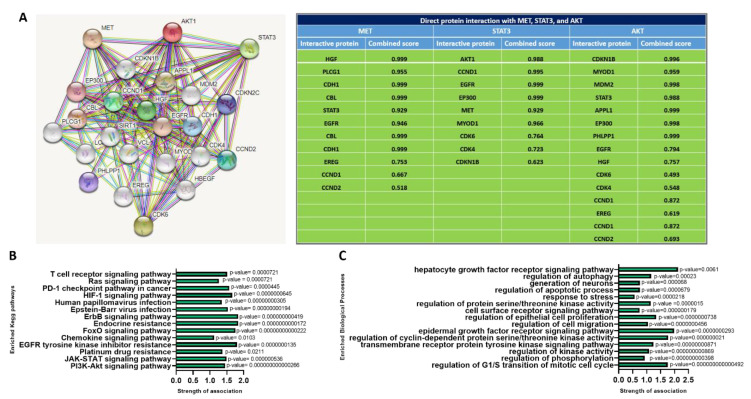
Protein–protein interaction (PPI) network visualization of MET, STAT3, and AKT. (**A**) Clustering network of MET, STAT3, and AKT interaction generated 24 nodes and 134 edges with an average local clustering coefficient of 0.486 and PPI enrichment of *p* < 10^−16^. Accompanying table shows the proteins interacting with MET, STAT3, and AKT representing the highest-scoring interacting link to 0.999 (**B**) The KEGG pathways and (**C**) biological processes associated with MET, STAT3, and AKT clustering networks.

The overexpressed genes in head and neck cancer were utilized to create the PPI network in order to discover the greatest number of proteins interacting with one another. The MET/STAT3/AKT clustering networks generated 24 nodes and 134 edges with an average local clustering coefficient of 0.486% and PPI enrichment of *p* < 10^−16^ (Figure 7A). In Figure 7A, accompanying a table of the network analysis, MET directly interacted with 11 proteins, with interactive scores of 0.518–0.999. The most profoundly interacting proteins with MET were STAT3, EGFR, HGF, CBL, and CDH1. STAT3 had close interactions with nine proteins, AKT, EGFR, CCND1, MET, CDK6, EP300, CDK4, CDKN1B, and MYOD1, with a combined score of 0.623–0.999. AKT had 15 total interactions, and the most closely interacting proteins were STAT3, CDKN1B, APPL1, MDM2, MYOD1, and EP300, with combined scores ranging from 0.493 to 0.999. According to the KEGG, the strongly associated pathways with MET/STAT3/AKT are the PI3K-AKT signaling pathway, Epstein–Barr virus infection, cell cycle, human papillomavirus infection, focal adhesion, EGFR tyrosine kinase inhibitor resistance, chronic myeloid leukemia, JAK-STAT signaling pathway, thyroid hormone signaling pathway, PD-L1 expression and PD1 checkpoint pathway in cancer, Wnt signaling pathway, VEGF signaling pathway, and platinum drug resistance pathway (Figure 7B). The topmost related biological processes in the MET, STAT3, and AKT network involved regulation of hepatocyte growth factor receptor signaling, epidermal growth factor receptor signaling, regulation of kinase activity, regulation of phosphorylation, regulation of cyclin-dependent protein serine/threonine kinase activity, response to stress, and regulation of the G1/S transition of the mitotic cell cycle (Figure 7C). 

### 2.7. In Silico Molecular Docking Simulations Predicted Strong Interactions between HNC018 and c-MET/STAT3/AKT

**Figure 8:**
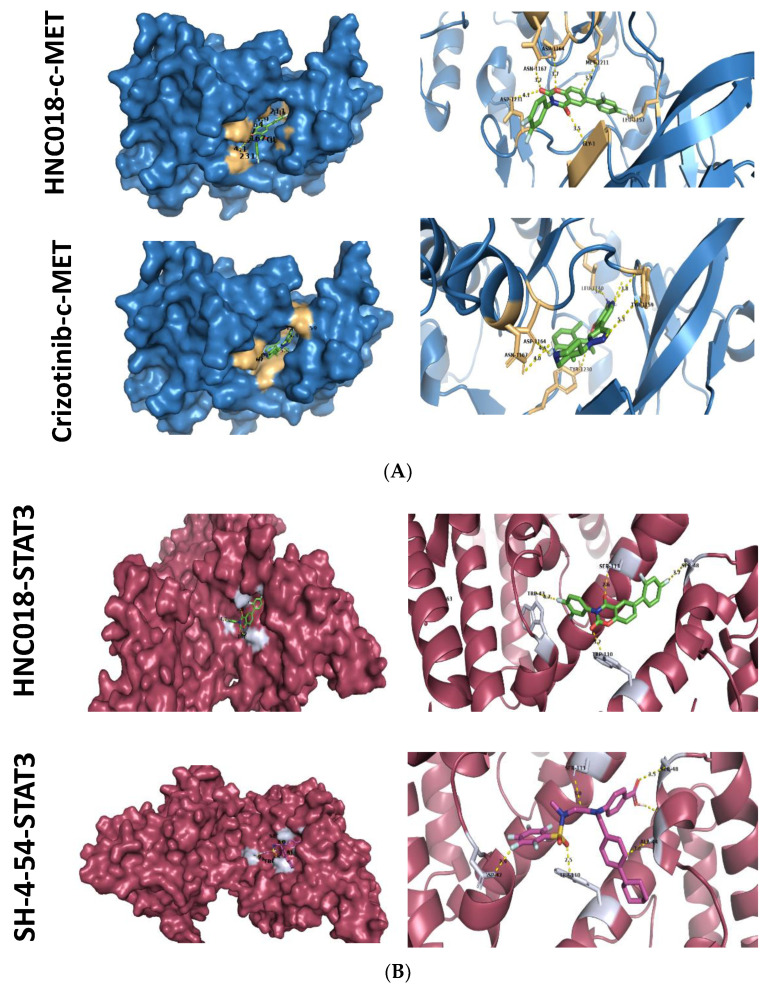

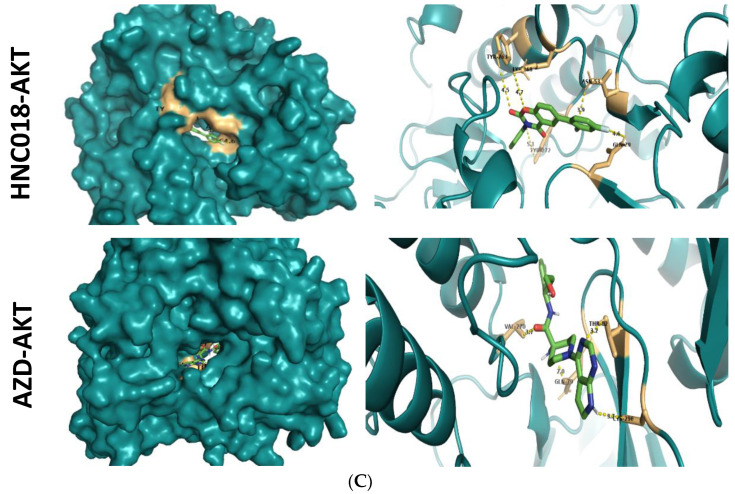
(**A**) Docking profile of c-MET with HNC018 and Crizotinib (a known inhibitor for HNSCC). A 3D structure of ligand–receptor interactions is shown in the left panel. The right panel shows the 2D representation of the interaction with ligands and the receptors in the binding pocket. (**B**) Docking profile of STAT3 with HNC018 and SH-4-54 (a known inhibitor for HNSCC). A 3D structure of ligand–receptor interactions is shown in the left panel. The right panel shows the 2D representation of the interaction with ligands and the receptors in the binding pocket. (**C**) Docking profile of AKT with HNC018 and AZD (a known inhibitor for HNSCC). A 3D structure of ligand–receptor interactions is shown in the left panel. The right panel shows the 2D representation of the interaction with ligands and the receptors in the binding pocket.

Following our bioinformatics analysis, we carried out a docking study to determine the possible interactions of HNC018 with c-MET, STAT3, and AKT (Figure 8A–C). The results reveal that the c-MET receptor is the promising protein target of HNC018, with a binding energy of −9.2 kcal/mol and a binding distance of 3.2 Å to the ASN1167 residue, 3.7 Å to the ASP1164 residue, and 3.9 Å to MET1211 of the receptor; this is comparable to the binding affinity of −8.8 kcal/mol obtained when c-MET was docked with Crizotinib, a known HNSCC inhibitor (Figure 8A and Table 3). Similarly, STAT3 interacts with HNC018 through four conventional H bonds with TRP43, TRP110, SER113, and SER48 in close proximity to 4.7 Å, 3.2 Å, 2.6 Å, and 4.7 Å, respectively, with a binding energy of −9.2 kcal/mol (Figure 8B, Table 4). The AKT–HNC018 complex is stabilized by four H-bonds with LYS268, ASN53, TRY263, and GLN79 in close proximity to 4.7 Å, 3.9 Å, 4.5 Å, and 4.6 Å, respectively, with a binding energy of 12.1 kcal/mol (Figure 8C and Table 5).

### 2.8. HNC018 Treatment Improved Cisplatin Sensitivity and Suppressed Cancer Cells-Initiated Tumor Growth In Vivo

**Figure 9:**
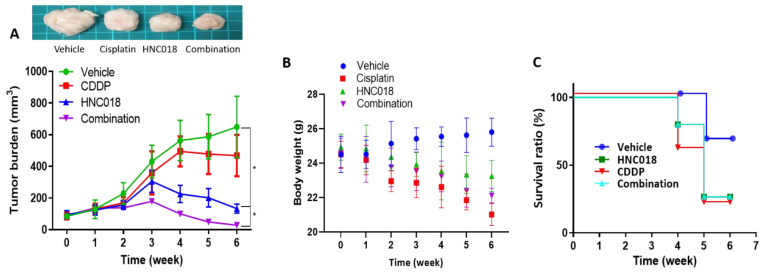
HNC018 treatment improved cisplatin sensitivity and suppressed cancer cells-initiated tumor growth in vivo. (**A**) An SAS cancer cells-bearing mouse model was established to evaluate HNC018′s anti-tumor efficacy. An average tumor volume versus time curve shows that HNC018 treatment significantly delayed tumor growth. (**B**) HNC018 treatment caused no significant changes in the body weights of the animals. The Kaplan–Meier survival curve (**C**) shows a non-significant survival ratio of animals treated with HNC018 alone or in combination with cisplatin compared to the control counterparts.

Using an SAS cells-bearing mouse model, we further examined the anti-HNSCC properties of HNC018 (Figure 9A). HNC018, cisplatin, and the combination of cisplatin and HNC018 (CDDP + HNC018) were compared to a vehicle control group. Since the purpose of this study was to evaluate HNC018′s potential in terms of anticancer treatment, we started with the lowest effective dose possible from the previously conducted trial on novel molecules in the treatment of HNSCC [3]. When compared to the control mice, mice treated with HNC018 (5 mg/kg, five times a week, intraperitoneal injection) showed considerably slower tumor growth (Figure 9A). More importantly, the addition of HNC018 re-sensitized the SAS cells towards cisplatin (CDDP) treatment, as demonstrated in the significantly reduced tumor burden in the combination group (Figure 9A). The average body weight was similar across the HNC018 and combination groups; however, it decreased somewhat in the CDDP-only group. (Figure 9B). The OS ratios for the HNC018 alone and combination groups were not statistically different from the untreated control group. (Figure 9C).

## 3. Discussion

Head and neck squamous cell carcinoma (HNSCC) is a disease with a high mortality rate. The c-MET pathway is engaged in the cross-talk, activation, and maintenance of other signaling pathways, which reduces the efficacy of a single-pathway blocking molecule. Because of extensive preclinical research indicating a link between HGF/c-MET signaling and cancer cell survival, clinical studies targeting the c-MET axis in HNSCC have been initiated [51]. The c-MET pathway and its therapeutic significance have been studied for over three decades. Overexpression of the c-MET receptor is frequently linked to poorer patient outcomes in HNSCC. Increased nodal metastases, cellular proliferation, and cell survival are responsible for this. Notably, c-MET contributes to treatment resistance by bypassing signals such as EGFR that are normally clinically blocked. MET inhibitors, on the other hand, have failed to demonstrate a significant improvement in patient outcomes [51]. Furthermore, c-MET-specific medicines must be developed and used in patients with adequate biomarkers [51]. The present state of knowledge about c-MET signaling and its effects on HNSCC requires a better understanding of how to overcome the overall survival rate of the patients because c-MET signaling is intertwined with other signaling pathways, and more robust and specialized therapeutics are possible, leading to better clinical outcomes. 

A population of cancer stem cells, which have unlimited potential for self-renewal and cause tumor regrowth if not entirely destroyed by therapy, support the growth of HNSCC [52]. Since the majority of these markers are cell surface proteins, they can be found using antibody-based techniques, such as Western blot. Therapy resistance is a major problem when treating cancer patients, as cancer stem cells are a subset of tumor cells and are resistant to anticancer treatments [53]. Cross-talk between c-MET networking and numerous other signaling molecules that are involved in specific physiological processes has developed. c-MET is activated in a paracrine manner by exerting its pleotropic effects through regulating a number of transmission cascades, including the Janus kinase/signal transducer and activator of transcription (JAK/STAT), phosphotidylinositol-3 kinase (PI3K/AKT). Inhibition of RTK pathways, which support proliferation and stemness in head and neck squamous cell carcinoma, can be partially offset by c-MET. As a result, treatment approaches that target c-MET and other receptor tyrosine kinases can be successful [54].

It is interesting to note that current research has linked c-MET to stem/progenitor cells originating from diverse adult normal tissues. In an adult mouse pancreas, 30% of label-retaining cells around the acini and ducts expressed c-MET, indicating them as a potential stem cell marker [55]. When c-MET^+^ liver cells were transplanted into the spleen or liver of mice with a liver injury, they formed stem cell colonies, migrated, and differentiated into liver parenchymal cells and cholangiocytes that are morphologically and functionally identical from their native counterparts [56]. Additionally, in the HNSCC cell lines, tumorsphere-derived exosomes promoted the expression of c-MET along with cisplatin resistance, and a tumor initiating ability [3], promoting the role of c-MET overexpression correlating to the maintenance of cancer stem cell properties in HNSCC and treatment failure [49]. This study further demonstrated that c-MET^+^ cardiac cells have stem cell qualities and could rebuild the infracted myocardium and increase ventricular function and long-term survival after HGF activation [57]. Based on these literature investigations, c-MET has been identified as a novel cell surface marker for CSC, at least in HNSCC, and also influences the chemoresistance in c-MET expressing HNSCC cells [58]. In addition, in this study, our drug, HNC018, has the ability to suppress the expression levels of a c-MET marker by reducing the tumorigenic properties associated with it. 

Here, the anti-tumorigenic characteristics of HNC018 against HNSCC were demonstrated using functional and bioinformatics data. In both in vitro and in vivo models, the HNC018 showed anti-tumor properties via downregulating the c-MET/STAT3/AKT signaling axis in HNSCC. First, we showed the frequent overexpression of the c-MET/STAT3/AKT in the Oncomine database, which correlates with poor overall survival in HNSC. The overexpression of c-MET correlates with the maintenance of the cancer stem cell properties in HNSCC and treatment failure [49]. The major factor influencing tumor growth, therapeutic response, and stemness is the formation of tumor heterogeneity, particularly the heterogeneity within the CSC population. Because of the plasticity of the CSC populations, cancerous cells with non-stem cell phenotypes can acquire stem cell characteristics as a result of genetic and epigenetic alterations [59,60,61].

Furthermore, by analyzing the ligand–receptor interactions, it was further established that kinases were the most likely targets for HNC018 based on predictions provided by an in silico Swiss target (Supplementary Figure S2). HNC018 is a superior ligand for c-MET, STAT3, and AKT than its conventional pharmacological inhibitors because of its close proximity and high binding affinities to receptors. The higher quantity of H-bonds, pi–sigma bonds, and pi–alkyl interactions may be the cause of HNC018’s strong binding to receptors. In the early stages of cancer, the idea of drug similarity can be very helpful in identifying possible treatment options [62]. 

Here, by taking into consideration the head and neck cancer tumor heterogeneity and its relationships to other prognostic markers, it is necessary to identify the innovative diagnostic markers. The combination of the molecularly targeted drugs has also demonstrated great efficacy in the treatment of head and neck squamous cell carcinoma patients, as eventually, HNSCC patients exhibit stemness and therapeutic resistance [59]. In this study, the combination therapy given with cisplatin and HNC018 has demonstrated the synergistic effects that help to overcome the therapy resistance of cisplatin in HNSCC patients. By downregulating the combined molecular targets, such as c-MET, STAT3, and AKT and their phosphorylated forms, the activation and overexpression of cancer stem cell-like cells is suppressed along with drug resistance signaling, hence improving the overall survival of the patients. 

Finally, we present preclinical data for HNC018 as a possible treatment drug for head and neck squamous cell cancer using the HNSCC cell line-carrying xenograft mice model. Comparing the HNC018-only group to the cisplatin-only group, the tumor growth was dramatically inhibited. Even though the HNC018 treatment has resulted in a reduction in tumor size, we still need to focus on improving the survival rate of the treated group. According to our in vitro testing, the combination of HNC018 with cisplatin had the greatest inhibitory effect on tumor development. The examination of treatment-related changes in mouse body weights (BWs) revealed that during the entire experiment, there was no substantial difference in the median BWs of mice treated with HNC018 alone or in combination with cisplatin.

**Figure 10:**
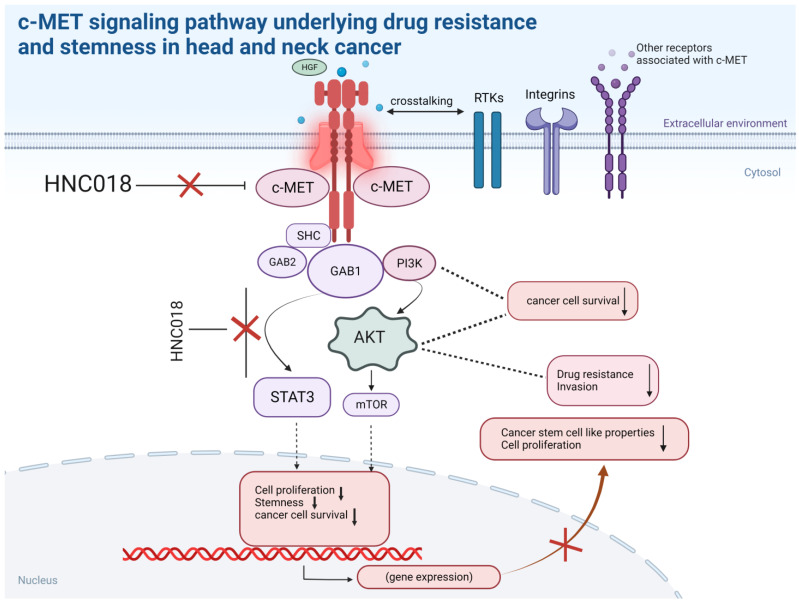
Schematic representation of the HNC018 targeting the c-MET pathway underlying drug resistance and stemness in head and neck squamous cell carcinoma.

Our findings demonstrate the therapeutic efficacy of HNC018 as a regulator and/or disruptor of various oncogenic pathways and significant drivers of head and neck squamous cell carcinoma and drug resistance, as shown in our schematic representation figure (Figure 10, image created with BioRender.com (accessed on 8 March 2022), and image is referenced from [63]). The results of the current study set the stage for further translational investigation and clinical application of HNC018 alone or as a synergistic enhancer of cisplatin anticancer efficacy in HNSCC-driven treatment-resistant or recurrent HNSCC cells, with the potential to increase patient survival rates.

## 4. Materials and Methods

### 4.1. Cell Culture and Reagents

The SAS cell line was received from the Japanese Collection of Research Bioresources (JCRB) Cell Bank, whereas the CAL27 cell line was obtained from the American Type Culture Collection (ATCC). The cells were maintained in accordance with the conditions specified for each culture. Cisplatin (CDDP) was acquired from SelleckChem (Hsinchu, Taiwan), whereas HNC018 was synthesized according to our prior investigation [39,64,65]. HNC018 (5 mM) and cisplatin (5 mM) stock solutions were produced in dimethyl sulfoxide (DMSO; Sigma Aldrich, St. Louis, MO, USA), and kept at −20 degrees Celsius.

### 4.2. Cell Viability Assay

Using an established procedure for sulforhodamine B (SRB) [66], the cell viability of SAS and CAL27 cells under various treatment conditions was determined. On a 96-well plate, 10,000 SAS and CAL27 cells were seeded per well, followed by 48 h of treatment with various concentrations of HNC018 (25, 12.5, 6.25, 3.125, 1.56, 0.78, 0.39 µM) and cisplatin (25, 12.5, 6.25, 3.125, 1.56, 0.78, 0.39 µM). After 48 h of treatment, the cells were rinsed with phosphate-buffered saline (PBS) and fixed with cold trichloroacetic acid for 60 min. The fixed cells were rinsed with ddH_2_O and stained with SRB (0.4% in 1% acetic acid) for 30 min. The unbound stain was removed using 1% acetic acid, and the plates were air-dried. The contents of the plates were dissolved for 15 min in a 20 mM Tris-based solution while agitation was maintained. The cell viability was assessed using a microplate reader to detect the absorbance at 515 nm (Molecular Devices, Sunnyvale, CA, USA).

### 4.3. Colony Formation Assay

SAS and CAL27 cells were seeded at 500 cells/well in Corning 6 well plates and treated with HNC018 at concentrations of 3.6 µM (SAS) and 3.1 µM (CAL27) for eight days. On the ninth day, the media were removed, and the colonies were stained and processed according to the established sulforhodamine B protocols (SRB) [66]. Using a Cell3iMager neo scanner, the colonies of the treated cells were quantified and compared with the control.

### 4.4. Generation of HNSCC Tumorspheres

The tumor-sphere generation assay was carried out in accordance with recognized procedures [67]. The SAS and CAL27 cells were seeded (2 × 10^4^ cells/well) and allowed to grow for 96 h in 6-well ultra-low attachment plates (Corning, Corning, NY, USA) in a serum-free medium consisting of Ham’s F12/Dulbecco’s modified Eagle medium (DMEM) (1:1), basic fibroblast growth factor (bFGF; 10 ng/mL, Pepro-Tech, Rocky Hill, NJ, USA), and 1% streptomycin/penicillin (100 U/mL, Hyclone, Logan, UT, USA). Suspended cells with a diameter >50 µm were considered a tumor-sphere and quantified using a Cell3iMager neo (CC-3000, Mitek Lab Co., Ltd., New Taipei City, Taiwan). 

### 4.5. Western Blot Analysis

The SAS and CAL27 cell lysates were analyzed for protein expression using SDS-PAGE with the Mini-Protein III system (Bio-Rad, Taipei, Taiwan) and transferred onto polyvinylidene difluoride membranes using the Trans-Blot Transfer System (Bio-Rad, Taipei, Taiwan), with or without treatment. The membranes were treated overnight at 4 °C with primary antibodies, followed by an incubation of 1 h at room temperature with the respective secondary antibodies. Enhanced chemiluminescence (ECL) detection kits were used to identify the immunoreaction signals, and images were acquired utilizing the U.V.P. BioDoc-It system (Upland, CA, USA).

### 4.6. Combination Median Effect Analysis

For quantitative evaluation of the nature of the HNC018 and cisplatin interaction, we utilized the isobologram approach based on the Chou–Talalay algorithm. The combination of cisplatin and HNC018 contained the given concentration ratio (as per the Figure 6). The CompuSyn program (ComboSyn Inc., Paramus, NJ, USA) was utilized to estimate the combination index (CI). CI = 1, CI < 1, or CI > 1 were interpreted as additive, synergistic, or antagonistic, respectively. Similarly, isobologram were also drawn based on the concentrations of HNC018 and cisplatin that induced the inhibition of viability; the drug combination data points that fall on the hypotenuse, within the right-angled triangle isobologram triangle, or outside the right-angled isobologram triangle were interpreted as an additive, synergistic, or antagonistic effect, respectively. 

### 4.7. Data Collection of Frequently Overexpressed Genes in Head and Neck Cancer through Various Databases

Currently, the Oncomine database (https://www.oncomine.org (accessed on 27 September 2021)) is the world’s largest database of oncogene chips and integrated data-mining platform for cancer gene knowledge mining, with 86,733 samples of cancer tissues and normal tissues, and 715 gene expression datasets having been collected [68,69]. On the basis of the Oncomine database, the classification of differential expressions of cancer types and their respective normal tissues was investigated. The Human Protein Atlas (HPA) (https://www.proteinatlas.org/ (accessed on 4 January 2022) provides large amounts of transcriptomic and proteomics data of specific human tissues, which is divided into cell, pathology, and the tissue atlas. The database offers information on 44 different normal tissue and organ cell-specific localization, along with 20 of the most common types of cancer [69,70]. Using the data from the HPA, immunohistochemical (IHC) expression maps of protein expression patterns in normal human tissues and tumor tissues were generated. The mRNA expression levels of the MET, STAT3, and AKT genes in head and neck cancer patients in the dataset of The Cancer Genome Atlas (TCGA) were analyzed using a gene expression profiling interactive analysis (GEPIA: https://gepia.cancer-pku.cn/ (accessed on 10 January 2022)) [71]. 

### 4.8. Clinical Data Mining with c-BioPortal Database and Correlation Analysis 

We analyzed the genetic changes (mutation, copy number variation) using the c-BioPortal tool (https://www.cbioportal.og/) (accessed on 11 January 2022) and performed altered and unaltered group comparisons of overexpressed genes in 10,953 cancer patients’ (10,967) samples from different cancer types [47,48]. The c-BioPortal tool is utilized for analyzing, exploring, and visualizing multidimensional cancer genomics data. We analyzed the MET’s correlation with STAT3 and AKT using the Tumor Immune Estimation Recourse Program (TIMER2.0) (https://timer.cistrome.org/) (accessed on 12 January 2022).

### 4.9. Clustering of Protein–Protein Interaction (PPI) Networks, Gene Ontology (GO), and Kyoto eEncyclopedia, Genes, and Genomes (KEGG) Pathway Analysis

Frequently overexpressed genes in head and neck cancer datasets were used to construct a PPI network by retrieving the interacting genes through an online search using the tool STRING, vers. 11.0 (https://string-db.org/ (accessed on 14 January 2022)) database. Functional protein partners in the PPI network, which regulate biological processes, were further identified. 

### 4.10. Molecular Docking Analysis

HNC018’s three-dimensional (3D) structure was depicted in Sybyl mol2 using the Avogadro molecular builder and visualization program, version 1.1.0. [72]. The structure was translated into the protein databank utilizing PyMOL Molecular Graphics System, version 1.2 rpre (Schrodinger, LLC, Palo Alto, CA, USA) (PDB). The PDB was queried for the 3D structure of the receptors and the crystal structures of c-MET (PDB:4GG5), STAT3 (PDB:4ZIA), and AKT (PDB:6S9W). Using AutoDock Vina, the PDB file formats of the ligands (HNC018 and Crizotinib) and receptors (c-MET, STAT3, and AKT) were converted to the AutoDock pdbqt format (vers. 0.8, The Scripps Research Institute, La Jolla, CA, USA) [73]. Pre-docking required the removal of water molecules, the addition of hydrogen atoms, and the formation of Kollman charges in the receptor. Molecular docking investigations were performed with AutoDock Vina software and the techniques outlined in our prior studies [3,74,75,76]. The docking data were reported as binding energy estimates (kcal/mol) for the optimal poses of ligand-receptor complexes involving hydrogen bonds and electrostatic and hydrophobic interactions. PyMOL software was utilized to illustrate the H-bond interactions, binding affinities, interacting amino acid residues, atoms bound to ligands and receptors, and 3D graphical representations of ligand-receptor complexes.

### 4.11. Animal Experiments

All the animal studies were conducted in accordance with Taipei Medical University’s Animal Research Ethics Committee’s requirements (Protocol LAC-2018-0414). We selecteed 6-week-old female NOD/SCID mice to show that parental SAS cells can start tumors by injecting 10^4^ cells under the skin into the right flank of each NOD/SCID mouse. The mice were randomly divided into 4 groups (n = 5) after the tumor had become palpable (about 2 weeks), including the vehicle control group, which received normal saline; the cisplatin group (CDDP, 1 mg/kg, i.p. injection, twice a week); the HNC018 group (HNC018 only, 5 mg/kg, i.p. injection, five times a week); and the combination group (CDDP+HNC018). For a period of six weeks, the tumor volume and body weight (BW) were tracked and measured once a week. The formula used to determine the tumor volume was tumor volume = length × width^2^/2 (unit mm^3^).

### 4.12. Data Analysis

A Student’s *t*-test analysis of the data was carried out using GraphPad Prism software version 6.04 for Windows (La Jolla, CA, USA). The results are presented as the mean +/− standard deviation (SD). Pearson’s correlation was used to assess the correlations of differentially expressed genes. The genetic alterations were calculated based on the c-BioPortal web tool instructions. The data from treatment groups were compared with the control using Student’s *t*-test, and the statistical significance was considered at *p* < 0.05 (*), *p* < 0.01 (**), *p <* 0.001 (***), and *p* < 0.0001 (****). 

## 5. Conclusions

In conclusion, our study of clinical HNSCC cohorts showed that dysregulation of the c-MET/STAT3/AKT signaling pathway was linked to the development of HNSCC, as well as its worse prognoses, resistance to treatment, progression of cancer stem cell-like phenotypes, and resistance to certain drugs. Through concurrent inhibition of the c-MET/STAT3/AKT signaling networks, HNC018, a novel multi-target small molecule, effectively inhibited the proliferation and oncogenic phenotypes of HNSCC. As a result, we propose that HNC018 may provide a new treatment option for HNSCC by functioning as a multi-target inhibitor of the c-MET/STAT3/AKT signaling networks of HNSCC.

## Figures and Tables

**Table 1:** Enriched genes in MET altered cohorts across a c-BioPortal database.

Gene	Cytoband	Altered Group	Unaltered Group	Log Ratio	*p*-Value	q-Value	Enriched in
*CAPZA2*	7q31.2	150 (38.96%)	34 (0.51%)	6.24	4.97 × 10^−167^	1.16 × 10^−162^	Altered group
*ST7*	7q31.2	159 (41.30%)	71 (0.94%)	5.46	5.67 × 10^−166^	6.62 × 10^−162^	Altered group
*TES*	7q31.2	137 (35.58%)	45 (0.59%)	5.92	9.57 × 10^−151^	7.46 × 10^−147^	Altered group
*WNT2*	7q31.2	142 (36.79%)	78 (0.90%)	5.35	1.57 × 10^−147^	9.17 × 10^−144^	Altered group
*ASZ1*	7q31.2	144 (37.40%)	82 (1.01%)	5.21	2.49 × 10^−145^	1.16 × 10^−141^	Altered group
*CFTR*	7q31.2	174 (44.85%)	269 (2.74%)	4.03	2.82 × 10^−142^	1.04 × 10^−138^	Altered group
*TFEC*	7q31.2	143 (37.05%)	91 (1.08%)	5.1	3.12 × 10^−142^	1.04 × 10^−138^	Altered group
*CTTNBP2*	7q31.31	168 (43.30%)	245 (2.58%)	4.07	1.23 × 10^−137^	3.60 × 10^−134^	Altered group
*CAV2*	7q31.2	127 (32.82%)	24 (0.44%)	6.23	3.34 × 10^−133^	8.67 × 10^−130^	Altered group
*CAV1*	7q31.2	125 (32.47%)	27 (0.51%)	5.98	2.23 × 10^−126^	5.21 × 10^−123^	Altered group
*MDFIC*	7q31.1-q31.2	112 (29.02%)	58 (0.69%)	5.39	8.27 × 10^−115^	1.76 × 10^−111^	Altered group
*ANKRD7*	7q31.31	117 (30.39%)	70 (0.88%)	5.12	5.17 × 10^−114^	1.01 × 10^−110^	Altered group
*FOXP2*	7q31.1	133 (34.37%)	174 (1.76%)	4.28	6.74 × 10^−113^	1.21 × 10^−109^	Altered group

**Table 2:** Enriched genes in MET unaltered cohorts across a c-BioPortal database.

Gene	Cytoband	Altered Group	Unaltered Group	Log Ratio	*p*-Value	q-Value	Enriched in
*WASH5P*	19p13.3	0 (0.00%)	164 (1.71%)	<−10	0.0014	0.00168	Unaltered group
*LINC01002*	19p13.3	0 (0.00%)	163 (1.70%)	<−10	0.00145	0.00175	Unaltered group
*C19ORF25*	19p13.3	0 (0.00%)	131 (1.35%)	<−10	0.00549	0.00628	Unaltered group
*STARD4-AS1*	5q22.1	0 (0.00%)	96 (1.33%)	<−10	0.00619	0.00705	Unaltered group
*LINC02200*	5q22.2	0 (0.00%)	100 (1.30%)	<−10	0.007	0.00793	Unaltered group

**Table 3:** Represents the ligand–receptor interacting atoms, binding affinities, and interacting distances of HNC018 and c-MET.

Ligand	Receptor	PDB ID	Binding Affinity (kcal/mol)	Bonding Length (Å)	Amino Acid Residue	Interaction
HNC018	c-MET	4GG5	−9.2	3.2	ASN1167	Hydrogen bond
				3.7	ASP1164	Pi-Anion
				3.9	MET1211	Pi-Alkyl
Crizotinib	c-MET	4GG5	−8.8	4.6	ASP1164	Hydrogen bond
				4.0	ASN1167	Hydrogen bond
				5.4	TRY1230	Pi-Pi stacked

**Table 4:** Represents the ligand–receptor interacting atoms, binding affinities, and interacting distances of HNC018 and STAT3.

Ligand	Receptor	PDB ID	Binding Affinity (kcal/mol)	Bonding Length (Å)	Amino Acid Residue	Interaction
HNC018	STAT3	4ZIA	−9.2	4.7	TRP43	Hydrogen bond
				3.2	TRP110	Hydrogen bond
				2.6	SER113	Hydrogen bond
				4.7	SER48	Hydrogen bond
SH-4-54	STAT3	4ZIA	−8.9	2.9	ASP42	Hydrogen bond
				2.5	TRP110	Hydrogen bond
				3.0	SER113	Hydrogen bond

**Table 5:** Represents the ligand–receptor interacting atoms, binding affinities, and interacting distances of HNC018 and AKT.

Ligand	Receptor	PDB ID	Binding Affinity (kcal/mol)	Bonding Length ( Å)	Amino Acid Residue	Interaction
HNC018	AKT	6S9W	−12.1	4.7	LYS268	Hydrogen bond
				3.9	ASN53	Hydrogen bond
				4.5	TRY263	Hydrogen bond
				4.6	GLN79	Hydrogen bond
AZD	AKT	6S9W	−9.9	3.7	VAL270	Hydrogen bond
				2.0	GLN79	Hydrogen bond
				3.2	THR82	Hydrogen bond

## Data Availability

The datasets generated and/or analyzed in this study are available on reasonable request.

## Supplementary Materials

The following supporting information can be downloaded at: https://www.mdpi.com/article/10.3390/ijms241210247/s1.
