# Supplementary material for: Multi-Omics Identification of Genetic Alterations in Head and Neck Squamous Cell Carcinoma and Therapeutic Efficacy of HNC018 as a Novel Multi-Target Agent for c-MET/STAT3/AKT Signaling Axis
Source: Int J Mol Sci. 2023 Jun 16;24(12):10247. doi: 10.3390/ijms241210247 (PMC10299157; doi:10.3390/ijms241210247)
Supplement: Supplementary file 1 [file ijms-24-10247-s001.zip › ijms-2415623-supplementary.pdf]

Supplementary Materials:

Article

Multi-omics identification of genetic alterations in head and neck squamous cell carcinoma and therapeutic efficacy of HNC018 as a novel multi-target agent for c-MET/STAT3/AKT signaling axis

Harshita Nivrutti Khedkar [1,2], Lung-Ching Chen [3,4], Yu-Cheng Kuo [5,6], Alexander T.H Wu [7,8,9,10,11,*] and Hsu-Shan Huang [1,2,11,12,13,*]

1. Ph.D. Program for Cancer Molecular Biology and Drug Discovery, College of Medical Science and Technology, Taipei Medical University, and Academia Sinica, Taipei 11031, Taiwan
2. Graduate Institute for Cancer Biology & Drug Discovery, College of Medical Science and Technology, Taipei Medical University, Taipei 11031, Taiwan
3. Division of Cardiology, Department of Internal Medicine, Shin Kong Wu Ho-Su Memorial Hospital, Taipei 11101, Taiwan
4. School of Medicine, Fu Jen Catholic University, New Taipei 24205, Taiwan
5. Department of Pharmacology, School of Medicine, College of Medicine, Taipei Medical University, Taipei 11031, Taiwan
6. School of Post-Baccalaureate Chinese Medicine, College of Chinese Medicine, China Medical University, Taichung 40402, Taiwan
7. The Ph.D. Program for Translational Medicine, College of Medical Science and Technology, Taipei Medical University, Taipei 11031, Taiwan
8. Taipei Heart Institute (THI), Taipei Medical University,Taipei, 11031, Taiwan
9. Clinical Research Center, Taipei Medical University Hospital, Taipei Medical University, Taipei 11031, Taiwan
10. International Ph.D. Program for Translational Science, College of Medical Science and Technology, Taipei Medical University, Taipei 11031, Taiwan
11. Graduate Institute of Medical Sciences, National Defense Medical Centre, Taipei 11490,Taiwan
12. School of Pharmacy, National Defense Medical Centre, Taipei 11490, Taiwan
13. Ph.D. Program in Drug Discovery and Development Industry, College of Pharmacy, Taipei Medical University, Taipei 11031, Taiwan

* Correspondence: chaw1211@tmu.edu.tw (A.T.H.W.); huanghs99@tmu.edu.tw (H.-S.H.);
Tel.: +886-2-2697-28 2035 (ext. 112) (A.T.H.W.); +886-2-6638-2736 (ext. 1377) (H.-S.H.)

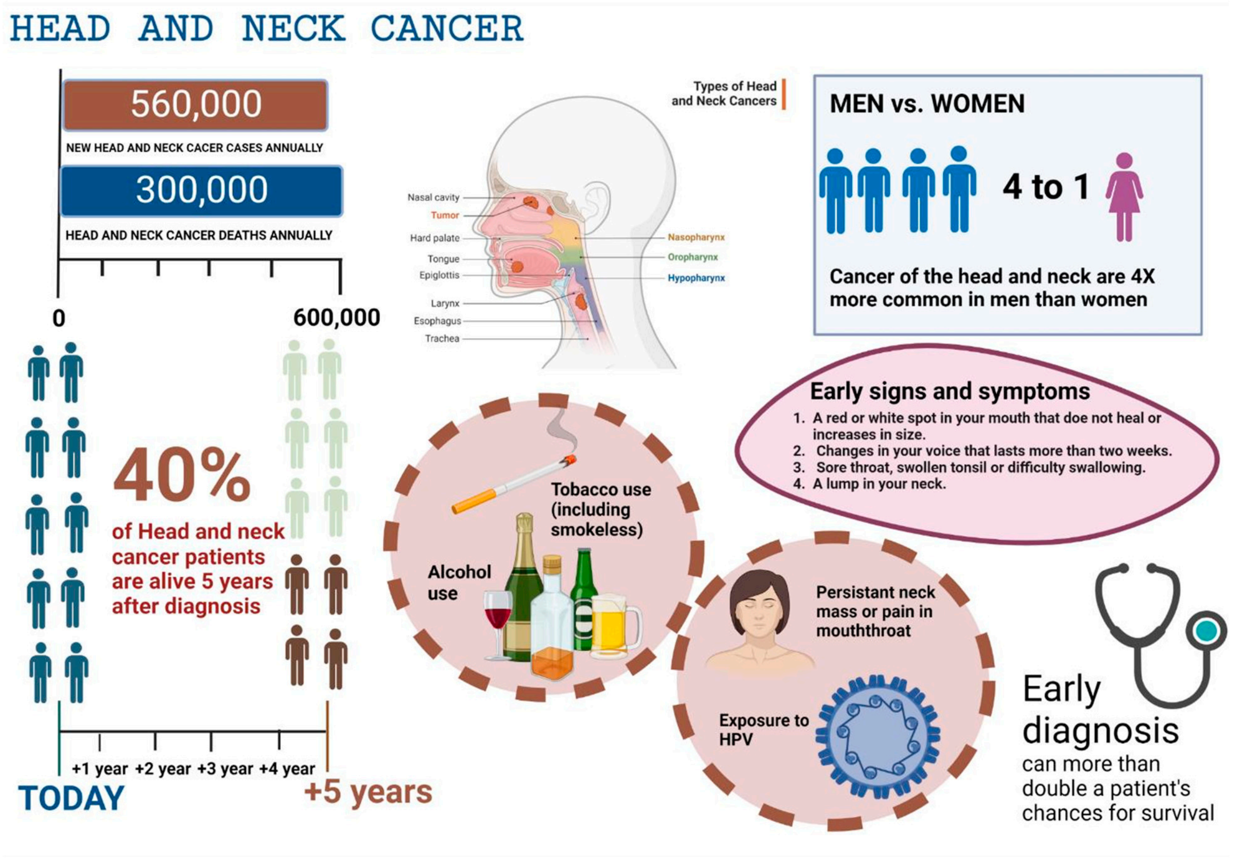

Supplementary Figure S1.: **An overview on head and neck cancer symptoms, causes and overall survival (Image created with BioRender.com).**

A

B

C

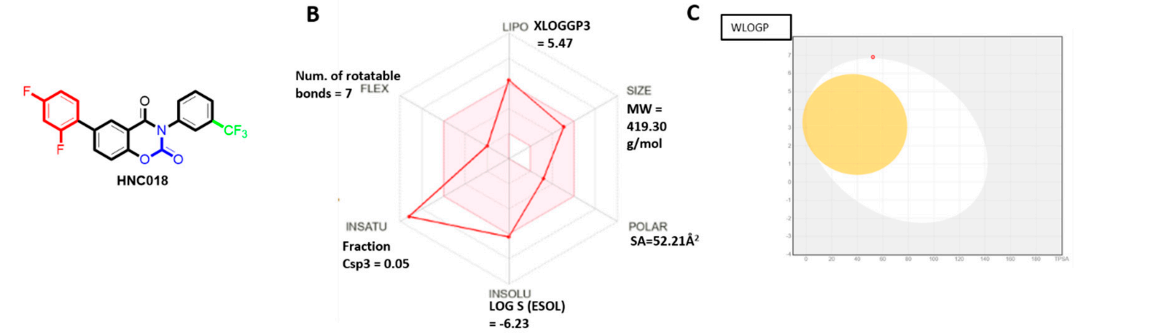

D

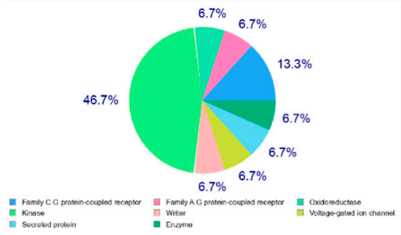

E

	Molecular Targets			Investigational Drugs			
Rank	P	CCLC	Target	P	CCLC	Targets	Mechanism Of Actions
1	0.27	57	RAF	0.48	58	TENALISIB	PI3K inhibitor
2	0.23	50	FGFR1	0.42	56	AMG706	VEGFR, PDGFR inhibitor
3	0.14	57	AKT	0.4	59	AMG319	PI3K inhibitor
4	0.13	56	TP53	0.39	59	AMG-458 (C-MET)	Met (c-Met) kinase inhibitor
5	0.12	58	EGFR	0.33	57	TAS120	FGFR inhibitor
6	0.12	58	CDK6	0.29	58	AZD-2014	mTOR inhibitor
7	0.11	58	MET	0.28	43	TOFACITINIB	JAK1, JAK3 inhibitor

P: Pearson's correlation coefficient. CCLC: Common cell lines count.

<u>**Supplementary Figure S2.:**</u> **The required standards for drug-likeness were met by HNC018.** In terms of lipophilicity, polarity, flexibility, solubility, saturation, and molecular weight, we discovered that HNC018 met the requirements of a good therapeutic candidate. (A) A schematic illustration of HNC018's structure. (B) Bioavailability radar displaying appropriate physicochemical spaces of HNC018's oral bioavailability. The pink area represents the optimal range of each property. (C) BOILED-EGG model for predicting the brain or intestine permeation of HNC018 (D) The pie graphic depicts the distribution of protein classes for prospective HNC018 medication candidates.(E) NCI synthesized compounds and conventional anticancer drug with comparable anticancer fingerprints and mechanistic link to HNC018.

Sr. no.	Properties	HNC018	Reference Value
1	Formula	C21H10F5NO3	-
2	M.W. (g/mol)	419.30 g/mol	150 - 500
3	Num. of rotatable bonds	3	0 - 9
4	H-bond ACC.	8	0 - 10
5	H-bond DON.	0	0 - 5
6	Molar Refractivity	98.44	40 ~ 130
7	TPSA (Å)	52.21	20 - 130
8	Fraction Csp3	0.05	0.25 ~ <1
9	Log S (ESOL)	-6.32	0 - 6
10	Drug-likeness	YES	
11	Lead-likeness	YES	
12	Bioavailability Score	0.55	> 0.1 (10%)

Supplementary Table S1.: Drug likeness profile of HNC018.

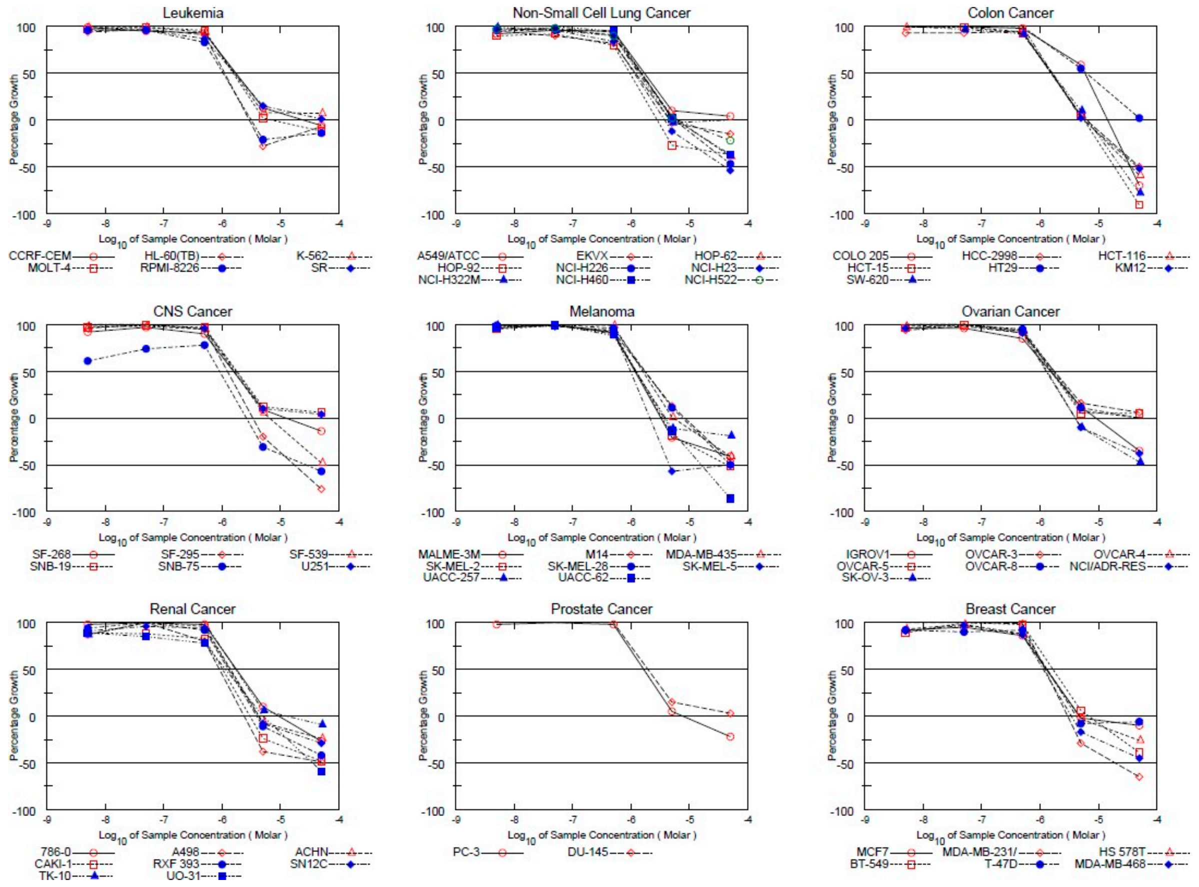

<u>Supplementary Figure S3.:</u> HNC018 demonstrated cytotoxic activities against the panel of National Cancer institute's cell lines. We tested HNC018 against the entire NCI-60 panel of Human tumor cell lines for anticancer properties. We discovered that HNC018 showed anti-proliferative activity against all of the NCI-60 cell line panels of breast, prostate, renal, ovarian, colon, melanoma, CNS, leukemia, and non-small cell lung cancers.

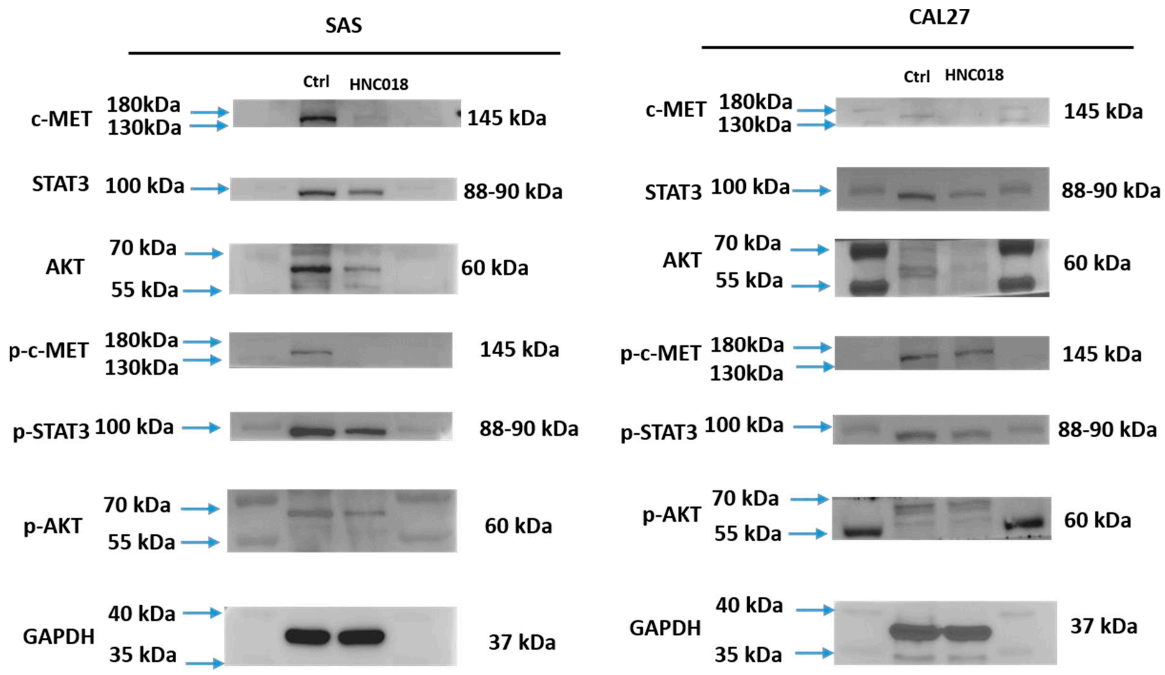

<u>Supplementary Figure S4.:</u> Western blot raw data of HNC018 treated SAS and CAL27 cell lines exhibitedlower expression levels of c-MET, p-c-MET, STAT3, p-STAT3, AKT, p-AKT.

Sr. No.	Antibody name	Lot Number	Company	Origin	Type	Dilution
1	p-c-met (Tyr 1234/1235)	(D26) XP® #3077S	CST Cell Signaling Technology	Rabbit	mAb (Monoclonal)	one is to one thousand
2	c-MET (N-Terminal)	19971-1-AP	Proteintech	Rabbit	pAb (Polyclonal)	one is to one thousand
3	p-STAT3-Y705	Cat No. AP0070	Abclonal	Rabbit	pAb (Polyclonal)	one is to one thousand
4	STAT3	(79D7) #4904	CST Cell Signaling Technology	Rabbit	mAb (Monoclonal)	one is to two thousand
5	p-AKT (Ser 473)	66444-1-Ig	Proteintech	Mouse	mAb (Monoclonal)	one is to two thousand
6	AKT (pan) (11E7)	#4685	CST Cell Signaling Technology	Rabbit	mAb (Monoclonal)	one is to one thousand
8	GAPDH	10494-1-AP	Proteintech	Rabbit	pAb (Polyclonal)	one is to five thousand

<u>Supplementary Table S2.:</u> List of antibodies used in this study.

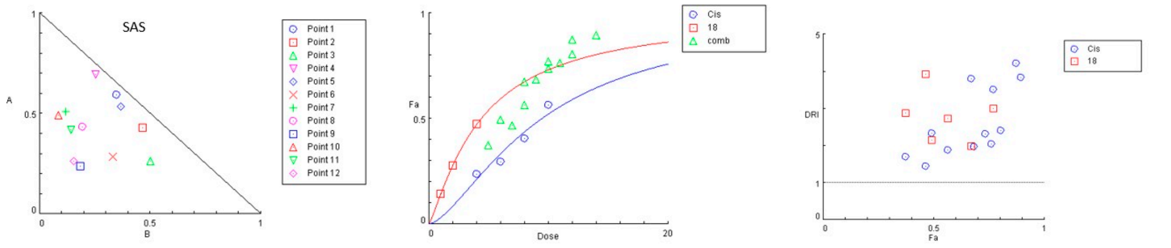

Combination therapy			
Cisplatin (μM)	HNC018 (μM)	Effect	CI
4	1	0.37487	0.93996
4	2	0.49367	0.89632
4	4	0.67247	0.76759
6	1	0.46656	0.94784
6	2	0.56505	0.90222
6	4	0.77209	0.61798
8	1	0.68454	0.62946
8	2	0.73589	0.62784
8	4	0.87403	0.4216
10	1	0.76256	0.57922
10	2	0.80514	0.55851
10	4	0.89415	0.41744

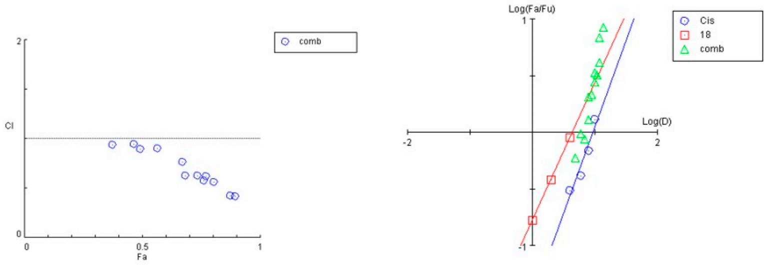

Supplementary Figure S5.: The combined action of HNC018 and Cisplatin against the SAS cell line was synergistic, using the Chou-Talalay-based algorithm for drug combination analysis.

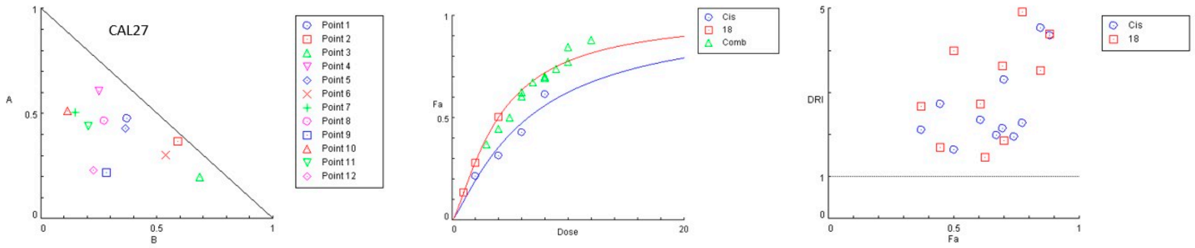

Combination therapy			
Cisplatin (μM)	HNC018 (μM)	Effect	CI
2	1	0.37102	0.84875
2	2	0.44588	0.96044
2	4	0.62661	0.88747
4	1	0.50278	0.85744
4	2	0.60628	0.79453
4	4	0.70044	0.84047
6	1	0.67249	0.65308
6	2	0.69416	0.73969
6	4	0.84725	0.50465
8	1	0.7395	0.63068
8	2	0.7733	0.64469
8	4	0.88203	0.45713

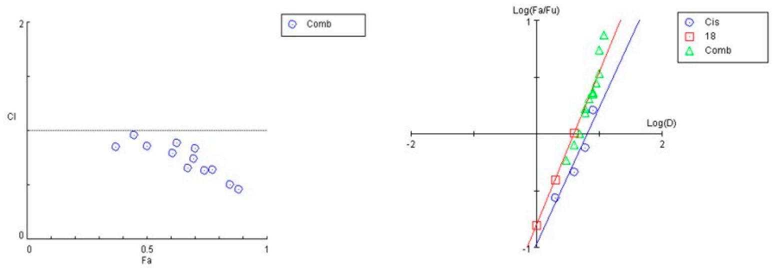

Supplementary Figure S6.: The combined action of HNC018 and Cisplatin against the CAL27 cell line was synergistic, using the Chou-Talalay-based algorithm for drug combination analysis.

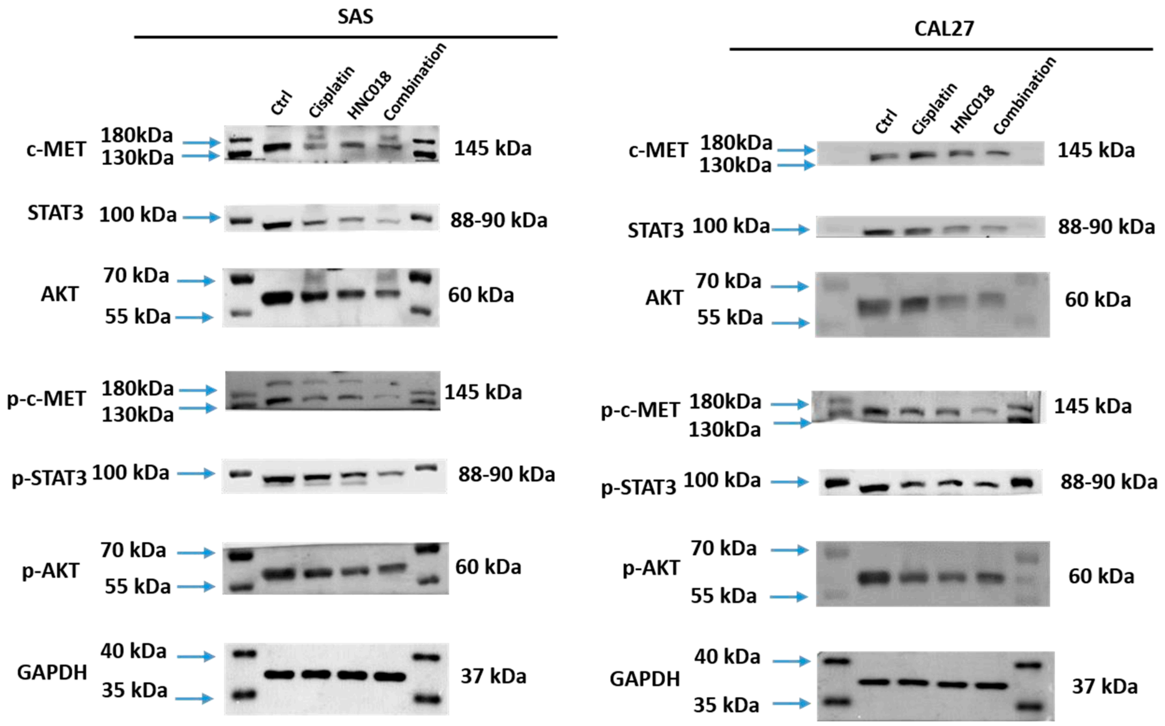

Supplementary Figure S7.: Western blot raw data demonstrating HNC018 and Cisplatin affect the expression level of c-MET, p-c-MET, STAT3, p-STAT3, AKT, p-AKT in HNSCC's SAS and CAL27 cell lines as compared to the control group.
